# Assessment of Bacteriological and Physicochemical Quality of Drinking Water from Source to Household Tap Connection in Nekemte, Oromia, Ethiopia

**DOI:** 10.1155/2019/2129792

**Published:** 2019-02-18

**Authors:** Gonfa Duressa, Fassil Assefa, Mulissa Jida

**Affiliations:** ^1^College of Natural and Computational Science, Wollega University, Nekemte, Ethiopia; ^2^College of Natural Science, Addis Ababa University, Addis Ababa, Ethiopia; ^3^Ethiopian Biotechnology Institute, Environmental Biotechnology Directorate, Addis Ababa, Ethiopia

## Abstract

In Ethiopia, access to improved water supply and sanitation has been very low and hence majority of the communicable diseases are associated with unsafe and inadequate water supply. Thus, the aim of this study was to assess physicochemical and bacteriological characteristics of water from sources to household connection in Nekemte town. A cross-sectional study was conducted from November 2015 to March 2016. Water samples were collected in triplicates from selected 30 sampling points from source, disinfection point, main distribution system tank, and household taps. All samples were analysed for bacteriological, chemical, and physical quality parameters using standard procedures. The results showed that temperature, pH, turbidity, total dissolved substances, and electrical conductivity of the water samples were varied between 16.9 and 22°C, 6.8–7.0, nil-12 NTU, 50–70 mg/l, and 40–46 *µ*S/cm, respectively. Phosphate and nitrate concentrations of the water samples also ranged between 0.65 and 1 mg/l and 2.2–6.5 mg/l, respectively. Free residual chlorine concentration in the majority of the water samples was less than 0.5 mg/l. All samples were positive for total coliform with counts ranging from 12 to 120 CFU/100 ml, whereas faecal coliforms were detected in only 37% of tap water samples. In general, the majority of the tested parameters were within the permissible range of both the WHO and Ethiopian drinking water standards. However, Fe, Mn, faecal coliforms, total coliforms, and temperature did not conform to both WHO and Ethiopian drinking water standards. Based on the results, we can conclude that water quality deterioration was both at the sources and in the supply networks. Hence, proper drainage, sewage disposal systems, and sufficient disinfection of water with chlorine are of prime importance to deliver safe drinking water to the residents of Nekemte town.

## 1. Introduction

The majority of population in developing countries has no access to clean water and any form of sanitation services. Consequently, millions of people are suffering from diseases related to water, sanitation, and hygiene, such as diarrhoea, skin diseases, and trachoma [1]. Unsafe water, inadequate sanitation, and poor hygiene are linked to 88% of diarrhoea cases worldwide [2]. Waterborne diseases are caused by the ingestion of water contaminated with human or animal faeces or urine containing pathogenic bacteria or viruses including cholera, typhoid, bacillary dysentery, adenoviruses, retroviruses, and other diseases. In addition, water derived from various sources may also contain dissolved inorganic and organic substances which could cause health problems to the community.

Poor water quality is considered as one of the manifestations of poverty in developing countries. Accordingly, improvement in drinking water supply, quality, and sanitation and reducing waterborne diseases have been major components of the sustainable development goal (SDG) programmes in goal 6 formulated by UN [3]. However, the effort of delivering safe water to the population has encountered several challenges. These are population growth, poor sanitation, and contamination of water sources with domestic wastes and industrial effluents [4, 5]. This shows that despite the worldwide efforts of delivering safe drinking water, the transmission of waterborne diseases is still a matter of major concern.

Ethiopia is confronted with poor sanitation and drinking water infrastructure [5]. About 52.1% of the population has been using unimproved sanitation facilities while 36% of them practiced open defecation [6]. It is estimated that more than 60% of the communicable diseases are due to poor environmental health conditions arising from unsafe and inadequate water supply with poor hygienic and sanitation practices [7]. Likewise, most health problems of children in the country are communicable diseases due to polluted water and improper sanitation.

The problems of contamination of urban water distribution system are diverse. The major sources of water contaminants are mostly wastes from improper sanitation and agricultural and other activities that make their way to the water distribution networks [8]. Furthermore, break in the distribution system, age and improper maintenance of the distribution system, and low level of chlorine usually compromise the integrity of the distribution system and quality of potable water.

Therefore, this requires proper protection of water supply from contamination and regular surveillance of water sources to reduce water-related diseases. Continuous examination of water quality analysis based on detection of indicator organisms is among the methods of assessing the hygienic condition of water [9]. Physicochemical parameters such as turbidity, pH, temperature, nitrate, and others are widely accepted as other critical water quality parameters for drinking water. These parameters either directly influence microbiological quality or affect disinfection efficiencies and human health.

Several studies carried out in Ethiopia on the physicochemical and bacteriological quality of drinking water from various sources showed that water sources were contaminated with pollution indicators such as faecal and total coliforms [4, 8–15]. These indicate that water-quality problems are rampant in water-delivery systems of the country. This necessitates establishment of sustainable monitoring and evaluation system of municipal water distribution systems. This study was, therefore, initiated to assess the status of drinking water quality from sources to households in Nekemte town.

## 2. Materials and Methods

### 2.1. Study Area

**Figure 1:**
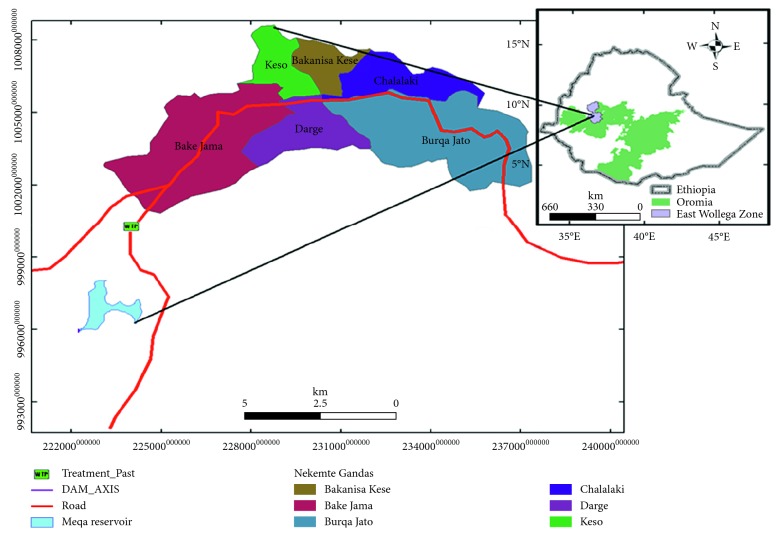
Map of the study site.

Nekemte is a market town and separate woreda in Western Ethiopia. It is located in the East Welega Zone of the Oromia Region. Nekemte has a latitude and longitude of 9°5′N 36°33′E/9.083°N 36.550°E and an elevation of 2,088 meters above sea level. The population of the town is estimated to be 110,688. The average temperature of the town is 17.6°C, and the average annual rainfall is 1988 mm. There are agricultural farms in and around the city which are irrigated with river water. These rivers receive numerous discharges of raw sewage, community refuse, and urban wastewater. Drinking water of the town comes from large reservoirs located in Diga Woreda, which is about 12 km away from the center of the town (Figure 1).

### 2.2. Study Design and Sampling Procedures

A cross-sectional study was conducted to assess the bacteriological and physicochemical quality of drinking water from source (untreated, before undergoing any treatment), disinfection point (treated, when it leaves the disinfection unit), main distribution system tank, and private tap of the town from September 2015 to March 2016. The samples were collected three times from each sampling site within 15 days interval.

A total of 30 triplicate water samples were collected from sampling points according to the WHO guidelines [16]. Of these, 27 were private taps distributed in 9 Kebeles (the smallest administrative unit of the town). Three different streets were selected purposely from each Kebele to include both the old and new areas. Then, three sites were selected by a simple random sampling method, and one household with tap water was selected randomly from each site. Written informed consent was obtained from each household to collect water sample and noted for second and third round sampling. Samples (250 ml) were collected in sterilized glass bottles rinsed thoroughly with nitric acid and sterilized distilled water. For the chlorinated water samples, about 2.5 ml sodium thiosulphate was added into each sampling bottle to stop the chlorination process during transportation [11]. Water samples were transported to Nekemte Water Supply and Sewerage Service Enterprise Laboratory and Oromia Regional Health Laboratory, Nekemte Laboratory, in a cold box containing ice freezer packs for physicochemical analysis and bacteriological count, respectively.

### 2.3. Sample Analysis

#### 2.3.1. Total and Faecal Coliforms Count

Total and faecal coliform counts were carried out by membrane filtration technique [15]. A sterilized pad dispenser was used to introduce the growth absorbent pads into the base of Petri dishes, and the growth pads were saturated with the Lauryl Sulphate Broth. 100 ml water sample was filtered using a membrane filter (0.45 *µ*m) in a vacuum filtration apparatus, and all the filters were transferred to the absorbent pad which was saturated with the broth. The Petri dishes were incubated at 37°C for 4 hr for resuscitation to recover physiologically stressed coliforms before incubation. Then after, plates for total coliform and thermotolerant coliform counts were incubated at 37°C and 44°C, respectively, for 24 hrs, and then colonies were counted and recorded [15].

#### 2.3.2. Analysis of Physicochemical Parameters

The physicochemical parameters were analysed by standard procedures. Physical parameters such as pH, temperature, and EC were measured using a portable digital pH meter, a thermometer, and an EC meter, respectively, at sites of sample collection. Turbidity of the samples was determined using a turbidity meter in the laboratory. The remaining water-quality parameters including nitrate, iron, manganese, phosphate, sulphate, and free residual chlorine, physical parameters, and TDS were determined following standard methods [15] using a DR 2400 spectrophotometer (Loveland, USA).

### 2.4. Data Analysis

The data were recorded, organized, and summarized in a Microsoft excel sheet. Data analysis was carried out by SPSS version 23. Descriptive statistics, one-way ANOVA (Duncan's multiple range test (DMRT)), and linear regression were employed to analyse the data at *p* < 0.05 significance level and 95% confidence interval.

## 3. Results and Discussion

### 3.1. Bacteriological and Physicochemical Characteristics of Water from Untreated Source, Reservoir, and Disinfection Point

The physiochemical characteristics of water samples collected from sources, disinfection, and distribution points are shown in Table 1. The results showed that average temperature records of the water samples range from 20.5 to 20.8 C without showing significant difference among the sources (Table 1). Similarly, earlier studies in Gondar zone [12] and Bahir Dar [17] reported a mean temperature of 21.3 C and 23.8 C, respectively. In tropics, the climate is characterized by high temperature and rainfall, and these factors might have contributed to the high temperature records of water samples from different cities of Ethiopia that did not meet the WHO standard of <15 C [18].

The pH of water samples was within the narrow range of 6.8–7.03 and did not vary significantly among sampling points. It remained within the recommended standard limits of 6.5–8.5 [18, 19]. The pH values of water obtained in this study are lower than the results of previous studies, i.e., the average basic pH records of various cities water sources, pH 7.6 at Akaki Kality, subcity of Addis Ababa [10]; pH 8.3 at Ziway [20]; and pH of 7.8 at Adama [14]. The variation could be due to geological conditions of the water sources.

The turbidity of water samples was within the range of 0–12 NTU with the highest record in untreated water from sources (Table 1). The average turbidity values of water sources were found to be significantly higher (*p* < 0.05) than that of treated water sources. Turbidity value of source water did not comply with both WHO and NGL standards [18, 19]. The TDS records of water samples in the present study were found to be below the recommended standard limits of both [18, 19].

Electrical conductivity in natural waters is the normalized measure of the water's ability to conduct electric current. This is mostly influenced by dissolved salts such as sodium chloride and potassium chloride. The highest EC (70 *μ*s/cm) was recorded from untreated water sources, while the lowest electrical conductivity was recorded from water samples obtained from disinfection (46 *μ*s/cm) and reservoir (45 *μ*s/cm) points. The EC records of water source from other cities of the country are much higher than the current study [11, 21]. These variations might originate from geological factors, agricultural activity, and the soil types of the study area.

Nitrate concentrations of the water samples from sources, disinfection point, and main distribution tank were 1.8 mg/1, 1.3 mg/1,and 1.3 mg/1, respectively. The phosphate concentrations were 1 mg/1, 0.6 mg, and 0.56 mg/1, respectively. The two ions concentrations in untreated water sources were significantly higher than the rest points. However, there were no variations between disinfection point and main distribution tank water sources both in nitrate and phosphate concentrations. Nevertheless, the nitrate records of the water sources were much less than the recommended limits of <50 mg/l in drinking water [16, 19]. This indicates that the nitrate and phosphate concentrations are not the problem of water in the study area. The nitrate content of water source samples measured in this study is less than the maximum values of 10.8 mg/l and 12.9 mg/l from source waters of Ziway town [20] and Bahr Dar town [17], respectively.

The free residual chlorine (FRC) content of water samples from disinfection point and main distribution tank were 0.23 mg/l and 0.28 mg/l, respectively. These values were less than the maximum concentration set by WHO. These values are slightly lower than the results reported at the treatment outlet of Ziway (0.79 mg/l) town [20] and recorded at Akaki Kality, subcity of Addis Ababa (0.67 mg/l) [10]. However, the FRC values were by far better than 0.03 mg/l recorded from the main distribution tank of Bahir Dar town [17].

The samples were also analysed for bacteriological quality, and our results showed that all water sources contained higher load of TC (Table 1). Likewise, FC was detected in water sources at all stages. In all cases, both TC and FC counts were above the recommended levels set by WHO standards [18]. However, significant reduction was observed for FC, which might be attributed to the treatment process. The degree of bacterial contamination of untreated source water in this study was found to be higher than that in the earlier studies [17, 22]. The difference between the current study and earlier works elsewhere in Ethiopia might be due to the variations of study season and socioeconomic characteristics of the communities residing in the study areas. In addition, the water sources of the current research area were surrounded by open defecation areas and many farming fields.

### 3.2. Bacteriological and Physicochemical Quality Analyses of Household Water Sources

#### 3.2.1. Bacteriological Quality of Household Tap Water

The highest TC count was recorded from tap water at sampling site 3 of Kebele 08, with 95 CFU/100 ml, followed by 78 CFU/100 ml at sampling site 1 of Kebele 08 (Table 2). The lowest TC count was found at sampling sites from 05 with 14 to 16 CFU/100 ml. Total coliform counts of the tap water were lower than water samples from the main distribution tank after treatment indicating that the treatment system significantly reduced microbial load of the water. On the contrary, more than 63% of the tap water samples did not show any FC count. The highest FC count of 22.5CFU/100 ml was recorded from sampling site 3 of Kebele 06, whereas no faecal coliform was detected from all sampling sites of Kebele 07 and 09 (Table 2).

Generally, faecal coliforms counts in household taps were lower than that recorded for the main distribution tank, except two sites from Kebele 08 and Kebele 05. The reduction of the TC and FC counts in household tap water samples could be attributed to free residual chlorine applied at the disinfection point of the treatment system. In general, 100% of the tap water samples did not meet the TC standard (1–10 CFU/100 ml) set by WHO, whereas about 37% of the samples failed to meet “safe water quality” with regard to FC criteria of 0 CFU/100/ml [16]. Similarly, a previous study had detected both TC and FC in all tap water samples of Bahir Dar city of Ethiopia [8].

#### 3.2.2. Physicochemical Quality of Household Tap Water

In this study, selected physical and chemical characteristics of household tap water which have public health implications were analysed to assess its status. The results of this study showed that the nitrate concentration was in the range of 2.2–6 mg/l with the lowest record from Kebele 02, site 01 and Kebele 04 site 03, and the highest record was from Kebele 01, site 02 (Table 2). This might be due to the over leaching of nitrate-containing organic wastes and from the use of fertilizers in the nearby agricultural fields. Our results are comparable to the average values of 0.3–7.0 mg/l from Ziway town [20], and less than nitrate content reported from Bahr Dar (9–30.1 mg/l) [17].

The phosphate concentration of the tap water samples was within 0.35–1 mg/l (Table 2). Majority (90%) of the water samples from household tap had higher phosphate concentration than disinfection point and main distribution tank water samples (0.6–0.56 mg/l) (Table 1), indicating that there is contamination of this ion in the supply network. The sulphate concentration of the tap water samples was within 11–26 mg/l, which was higher than the levels recorded from source, disinfection point, and main distribution tank water samples (4–14.5 mg/l) (Table 1).

All water samples from households contained less than 0.5 mg/l FRC except all sites from Kebele 03. Furthermore, two site samples did not contain FRC. All taken together, more than 15.2% of the water samples was found to be inferior to the proper water treatment standard using chlorine set by WHO [18] and ESA [19]. In general, this indicates that there is limitation in the water chlorination system in the town. Similar studies showed that 37.5% and 95.7% of tap water samples from tap water distribution systems in Ziway [20] and Bahr Dar towns [17] contained lower FRC than the recommended limits.

Our results showed that the temperature of the tap water samples ranged between 16.8°C and 22.1°C without showing any significant difference among the sites (Table 3) and hence did not meet the WHO standard of <15°C [18]. Likewise, similar earlier studies [8, 12] reported higher temperature values in drinking water from different Ethiopian cities. The pH of tap water samples varied from 6.6 to 7.0 (Table 3) without any significance among the sampling points. In general, the pH values of drinking water samples from source to private tap were in the recommended range of 6.5–7.8 (WHO 2006; ESA 2013). Similarly, previous studies showed that the pH values of drinking water in various cities of the country were within the recommended range [10, 14, 20, 23].

The turbidity of the tap water samples was in the range of 0.1–1.7 NTU without significant differences among the sampling sites. The turbidity of all tap water samples was significantly lower than that of the source water (Tables 1 and 3), and all of them were below 5NTU, a maximum acceptable value of WHO [16] and ESA [18]. Compared to the results of other similar studies from different towns of the country, turbidity records of tap water in Nekemte is very low [8, 10, 14, 20].

The TDS (37–46.5 mg/l) measurements of the tap water samples did not show significant (*p* < 0.05) differences from the water sources and water main distribution tanks showing that there is no external factor that could contribute to the increase or decrease of these parameters in the tap water distribution system. The TDS values of tap water samples fell below the maximum acceptable standard of 600 mg/l [16]. Similarly, Yasin et al. [11] had obtained lower TDS (524.7) in Serbo town tap water. In contrast, high TDS values were recorded from tap water of Debre Zeit town [23]. The electrical conductivity (EC) measurements of water samples were found to be in the range of 58–70 *μ*S/cm. These results were much lower than those of the research conducted in Debre Ziet [23] and Serbo towns [11].

## 4. Conclusions

In this study, the physicochemical and bacteriological quality of water samples from untreated source, main distribution system (main distribution tank), and tap water supply systems of Nekemte town were analysed, and majority of them were within the permissible range of both WHO and Ethiopian drinking water standards. However, Fe, Mn, FC, TC, and temperature did not meet the standards. About 85% of tap water samples analysed showed FRC values less than the standard set by WHO. This indicates that there was insufficient chlorination in the treatment system. All water samples from both the sources and household taps were contaminated by TC and FC. On the contrary, the majority of water samples from household taps were not contaminated with FC. This implies that water contamination is both at the source and in the supply network. Moreover, the presence of water-quality indicator bacteria might be associated with poor waste disposal systems and management of water sources. Therefore, there is a need to design an efficient waste disposal system and a catchment area management system around the water sources. In addition, regular drinking water-quality assessment of the source, main distribution tanks, distribution systems, and pipes should also be employed to ensure that the water is safe for human use. The present study was limited to assessing bacteriological and physical-chemical parameters from source to household tap connections of the water supply system in dry season. Thus, a similar study should be conducted during the wet season of the year. In addition, further study should consider additional water-quality parameters such as heavy metals and their source.

## Figures and Tables

**Table 1:** Physicochemical analysis of source, reservoir, and disinfection points water samples.

Parameters	Sources	Disinfection point	Main distribution tank	Tap water
N	3	3	3	81
Temp ( C) ± SE	20.5 ± 0.5^a^	20.8 ± 0.4^a^	20.8 ± 0.5^a^	20.9 ± 1.0^a^
pH ± SE	6.82 ± 1.7^a^	6.87 ± .01^a^	7.03 ± 01^a^	6.73 ± 0.1^a^
Turbidity (NTU) ± SE	12 ± 2^a^	Nil	Nil	0.89 ± 0.5^b^
TDS (mg/l) ± SE	47.8 ± 2.6^a^	36 ± 2^b^	32 ± 1^b^	21.0 ± 2.8^c^
EC (*µ*S/cm) ± SE	70 ± 2^a^	46 ± 1^b^	45 ± 0.00^b^	65.5 ± 4.5^a^
NO_3_^−^ (mg/l) ± SE	1.8 ± 0.2^b^	1.3 ± 0.3^b^	1.3 ± 0.2^a^	3.6 ± 1.1^a^
PO_4_^−3^ (mg/l) ± SE	1 ± 0.52^a^	0.6 ± .26^a^	0.56 ± .21^a^	0.75 ± 0.14^a^
FCR (mg/l) ± SE	0.20 ± 0.1^a^	0.23 ± .01^a^	0.28 ± .01^a^	0.21 ± 0.23^a^
S0_4_^−2^ (mg/l) ± SE	4 ± 1.7^b^	14 ± 3.6^a^	14.5 ± 3.5^a^	17.8 ± 3.4^a^
Colour (mg/) ± SE	91 ± 8.5^a^	3 ± 2.6^d^	Nil ± 0^d^	0.0
Mn^+2^ (mg/l) ± SE	1 ± 0.1^a^	0.3 ± .12^b^	0.2 ± 0.01^b^	1.6 ± 0.3^a^
Fe^+2^ (mg/l)	1.19 ± 0.1^a^	0.19 ± 0.0^c^	0.18 ± 0.0^c^	0.67 ± 0.2^b^
TC (CFU/100 ml) ± SE	120^a^	109^ab^	100^ab^	38 ± 15.7^c^
FC (CFU/100 ml) ± SE	TNT	12^a^	5.5^b^	1.7 ± 2.9^c^

Data are average of triplicates; numbers followed by the same letter superscripts in the same row do not vary significantly by Duncan's multiple range test at *p* < 0.05; SE: standard error; TNT: too numerous to count.

**Table 2:** Bacteriological and chemical parameters analysis of household tap water.

Kebeles	Site	Fe^+2^ (mg/l)	Mn^+2^ (mg/l)	S0_4_^−2^ (mg/l)	P0_4_^−3^ (mg/l)	N0_3_^−^ (mg/l)	Cl_2_ (mg/l)	TC (CFU/100 ml)	FC (CFU/100 ml)
01	S1	0.5 ± 0.02^b^	2.4 ± 0.30^a^	17.5 ± 1.5^ab^	0.75 ± 0.15^ab^	3.61 ± .49^ab^	0.06 ± 0.00^a^	20 ± 1.5^de^	0
	S2	0.50 ± 0.22^b^	1.50 ± 0.40^b^	19.5 ± 4.5^a^	0.85 ± 0.15^a^	6 ± 0.55^a^	0.12 ± 1.15^c^	21 ± 1.1^de^	2 ± 0.2^c^
	S3	0.60 ± 0.02^b^	1.51 ± 0.15^b^	22 ± 1.0^a^	0.90 ± 0.00^a^	4.4 ± 0.60^a^	0.20 ± 3.05^c^	20 ± 0.5^de^	0

02	S1	0.67 ± 0.03^b^	1.30 ± 0.05^b^	16 ± 2.0^b^	0.75 ± 0.05^ab^	2.2 ± 1.1^b^	0.12 ± 0.07^c^	40 ± 1.7^cd^	3 ± 0.5^bc^
	S2	0.50 ± 0.01^b^	1.30 ± 0.17^b^	11 ± 3.0^c^	0.80 ± 0.30^a^	3.2 ± 1.75^b^	0.04 ± 0.36^c^	23 ± 1.13^de^	0
	S3	0.42 ± 0.02^b^	1.30 ± 0.10^b^	18 ± 1.0^a^	0.85 ± 0.04^a^	3.1 ± 2.10^b^	0.07 ± 0.41^c^	41 ± 2.5^cd^	4 ± 0.4^bc^

03	S1	1.30 ± 0.2^a^	1.20 ± 0.10^b^	18.5 ± 0.5^a^	0.85 ± 0.05^a^	3 ± 1.35^b^	0.8 ± 0.15^a^	34 ± 1.9^d^	0
	S2	1.10 ± 0.34^a^	1.85 ± 0.06^a^	17 ± 1^ab^	0.51 ± 0.23^b^	3 ± 0.8^b^	0.9 ± 0.12^a^	65 ± 1.5^bc^	5 ± 0.6^bc^
	S3	0.70 ± 0.12^b^	1.75 ± 0.15^a^	14 ± 1^ab^	0.90 ± 0.05^a^	3 ± 0.60^b^	0.8 ± 0.12^a^	55 ± 5.7^c^	2 ± 0.2^bc^

04	S1	0.78 ± 0.19^b^	1.70 ± 0.10^a^	26 ± 2^a^	0.7 ± 0.20^ab^	3.6 ± 0.5^ab^	0.1 ± 0.02^c^	52 ± 5.0^c^	0
	S2	0.51 ± 0.01^b^	1.50 ± 0.15^b^	19 ± 1.1^a^	0.75 ± 0.05^ab^	5.7 ± 0.40^a^	0.3 ± .05^b^	35 ± 1.4^d^	0
	S3	0.51 ± 0.07^b^	1.15 ± 0.10^b^	14 ± 0.7^b^	0.71 ± 0.01^ab^	2.2 ± 0.20^b^	0.0	23 ± 5.7^de^	5 ± 0.5^bc^

05	S1	0.73 ± 0.17^b^	1.15 ± 0.06^b^	18.5 ± 2.5^a^	0.75 ± 0.15^ab^	4.4 ± 0.06^a^	0.01 ± 0.06^c^	15 ± 2.6^e^	0
	S2	0.73 ± 0.11^b^	1.75 ± 0.05^a^	16.5 ± 4.0^b^	0.85 ± 0.05^a^	3.5 ± 0.20^ab^	0.01 ± 0.00^c^	14 ± 6.4^e^	2 ± 0.12^bc^
	S3	0.64 ± 0.02^b^	2.00 ± 0.15^a^	16.5 ± 1.5^b^	0.80 ± 0.10^a^	3.4 ± 0.40^b^	0.51 ± 0.03^b^	16 ± 6.5^e^	5.5 ± 0.5^bc^

06	S1	0.41 ± 0.07^b^	2.00 ± 0.11^a^	16 ± 3^b^	0.65 ± 0.15^b^	3.5 ± 0.55^ab^	0.35 ± 0.27^b^	22 ± 1.50^de^	0
	S2	0.42 ± 0.01^b^	2.50 ± 0.30^a^	20.5 ± 0.5^a^	0.65 ± 0.06^b^	3 ± 1.15^b^	0.02 ± 0.10^c^	28 ± 0.7^de^	2.5 ± 0.3^bc^
	S3	0.85 ± 0.02^a^	1.80 ± 0.15^a^	23.5 ± 0.6^a^	0.85 ± 0.09^a^	3 ± 0.45^b^	0.40 ± 0.02^b^	23 ± 9.3^de^	0

07	S1	0.75 ± 0.05^b^	1.50 ± 0.01^b^	25 ± 1^a^	0.75 ± 0.21^ab^	3.75 ± 0.5^ab^	0.03 ± 0.01^c^	26 ± 0.8^d^	0
	S2	0.74 ± 0.15^b^	1.30 ± 0.1^b^	18.5 ± 0.1^a^	1.00 ± 0.30^a^	3 ± 0.36^b^	0.45 ± 0.25^b^	30 ± 1.6^d^	0
	S3	0.90 ± 0.01^a^	1.75 ± 0.10^a^	16 ± 1^b^	0.86 ± 0.01^a^	2.4 ± 0.26^b^	0.04 ± 0.01^c^	41 ± 7.6^cd^	0

08	S1	0.85 ± 0.25^a^	1.75 ± 0.17^a^	17.5±2.7^ab^	0.65 ± 0.06^b^	2.75 ± 0.20^b^	0.01 ± 0.00^c^	40 ± 1.15^cd^	0
	S2	0.75 ± 0.27^b^	1.65 ± 0.10^a^	13.5 ± 0.5^c^	0.35 ± 0.20^b^	2.5 ± 0.15^a^	0.20 ± 0.02^c^	60 ± 4.6^bc^	22.5 ± 0.8^a^
	S3	0.51 ± 0.47^b^	1.50 ± 0.1^a^	14 ± 2^b^	0.71 ± 0.09^ab^	4.4 ± 0.05^a^	0.03 ± 0.16^c^	95 ± 2.8^a^	0

09	S1	0.50 ± 0.15^b^	1.35 ± 0.01^b^	17.5 ± 2.5^a^	0.70 ± 0.04^ab^	4.25 ± .55^a^	0.0	78 ± 1.5^b^	0
	S2	0.72 ± .25^b^	1.49 ± 0.25^b^	19 ± 1^a^	0.50 ± 0.11^b^	5.5 ± 1.40^a^	0.01 ± 0.00^c^	60 ± 1.70^bc^	0
	S3	0.53 ± 0.06^b^	1.55 ± 0.06^b^	16 ± 2^a^	0.90 ± 0.05^a^	5.75 ± 0.20^a^	0.01 ± 0.00^c^	62 ± 2.8^bc^	0

*Note*. Data are average of triplicates; SE: standard error; TC: total coliform; FC: faecal coliform; S: sampling site; CFU: colony forming unit; numbers indicated by the same letter superscripts do not vary significantly by Duncan's multiple range test at *p* < 0.05.

**Table 3:** Physical analyses of household water sources.

Kebeles	Site	pH ± SE	Turbid (NTU) ± SE	Temp (°C) ± SE	TDS (mg/l) ± SE	EC (*µ*S/cm) ± SE
01	S1	6.7 ± 0.10^a^	0.9 ± 0.28^ab^	22.1 ± 0.87^a^	45.5 ± 0.52^a^	70.1 ± 0.90^a^
	S2	6.85 ± 0.03^a^	1.70 ± 0.80^a^	21.4 ± 1.15^a^	46.7 ± 0.46^a^	69 ± 1.15^a^
	S3	6.78 ± 0.08^a^	0.5 ± 0.05^ab^	21.8 ± 0.35^a^	45 ± 0.30^a^	74.2 ± 3.05^a^

02	S1	6.75 ± 0.02^a^	0.7 ± 0.40^ab^	16.8 ± 5.68^a^	45.7 ± 1.61^a^	69.1 ± .34^a^
	S2	6.70 ± 0.13^a^	0.35 ± 0.03^ab^	22 ± 1^a^	40.6 ± 0.69^a^	63.35 ± 1.32^a^
	S3	6.75 ± 0.12^a^	0.45 ± 0.05^ab^	21.75 ± 0.86^a^	45 ± 1.27^a^	64.25 ± 2.75^a^

03	S1	6.5 ± 0.05^a^	0.9 ± 0.07^ab^	21 ± 0.50^a^	40 ± 1.03^a^	68.3 ± 4.20^a^
	S2	6.6 ± 0.01^a^	0.75 ± 0.50^ab^	22 ± 0.01^a^	40 ± 0.8^a^	62.45 ± 6.05^a^
	S3	6.7 ± 0.01^a^	0.75 ± 0.70^ab^	22 ± .35^a^	37.5 ± 1.73^a^	62.75 ± 0.37^a^

04	S1	6.7 ± 0.10^a^	0.7 ± 0.20^ab^	20.75 ± 1.5^a^	41 ± 0.28^a^	58.25 ± 3.5^ab^
	S2	6.72 ± 0.09^a^	1.6 ± 0.80^a^	20.9 ± 1.09^a^	38 ± 0.79^a^	61.15 ± 1.05^a^
	S3	6.75 ± 0.05^a^	1.5 ± 1.15^a^	20.8 ± 4^a^	40 ± 0.61^a^	58.9 ± 0.40^ab^

05	S1	6.7 ± 0.11^a^	1.2 ± 0.80^a^	20.6 ± 0.46^a^	37 ± 0.69^a^	70 ± 0.10^a^
	S2	6.6 ± 0.11^a^	1.7 ± 0.80^a^	21.4 ± 0.60^a^	43 ± 3^a^	70.25 ± 1.3^a^
	S3	6.75 ± 0.10^a^	0.4 ± 0.02^ab^	20.1 ± 0.32^a^	44.5 ± 0.57^a^	68.12 ± 1.3^a^

06	S1	6.7 ± 0.02^a^	1.6 ± 0.85^a^	20.2 ± 2^a^	43.5 ± 0.3^a^	62.15 ± 0.07^a^
	S2	6.6 ± 0.15^a^	1.5 ± 1.10^a^	20 ± 1.75^a^	42.5 ± 0.23^a^	62.35 ± 0.01^a^
	S3	6.7 ± 0.02^a^	2.5 ± 0.20^a^	21.5 ± 6.5^a^	45 ± 1.05^a^	64.95 ± 0.01^a^

07	S1	7.0 ± 0.10^a^	0.5 ± 0.25^ab^	21 ± 0.9^a^	40.5 ± 0.57^a^	58.35 ± 0.80^ab^
	S2	7.0 ± 0.04^a^	0.5 ± 0.15^ab^	21.5 ± 0.6^a^	45 ± 1^a^	64.65 ± 0.5^a^
	S3	6.8 ± 0.04^a^	0.15 ± 0.05^ab^	20 ± 0.37^a^	44 ± 1.15^a^	58.35 ± 1.4^ab^

08	S1	6.7 ± 0.63^a^	1.2 ± 0.60^a^	20 ± 0.32^a^	41.5 ± 1.5^a^	71 ± 1.15^a^
	S2	6.7 ± 0.10^a^	0.7 ± 0.35^ab^	21 ± 0.51^a^	46 ± 1.15^a^	70 ± 0.65^a^
	S3	6.8 ± 0.15^a^	0.1 ± 0.05^ab^	21 ± 0.72^a^	45 ± 1^a^	70 ± 1.15^a^

09	S1	6.7 ± 0.10^a^	0.2 ± 0.03^ab^	21 ± 1.23^a^	43.5 ± .57^a^	69.5 ± 0.57^a^
	S2	6.7 ± 0.01^a^	0.5 ± 0.01^ab^	21 ± 1.35^a^	46 ± 1.15^a^	69.5 ± 3.46^a^
	S3	6.8 ± 0.06^a^	0.5 ± 0.025^ab^	22 ± 0.86^a^	46.5 ± 0.57^a^	58.45 ± 1.22^ab^

*Note*. Data are average of triplicates; SE: standard error; numbers indicated by the same letter superscripts within the same column do not vary significantly by Duncan's multiple range test at *p* < 0.05.

## Data Availability

All data supporting the findings of this study are included in the paper; however, details of the full data may be accessed by contacting the corresponding author.
